# Genome-Wide Association Analysis With a 50K Transcribed Gene SNP-Chip Identifies QTL Affecting Muscle Yield in Rainbow Trout

**DOI:** 10.3389/fgene.2018.00387

**Published:** 2018-09-19

**Authors:** Mohamed Salem, Rafet Al-Tobasei, Ali Ali, Daniela Lourenco, Guangtu Gao, Yniv Palti, Brett Kenney, Timothy D. Leeds

**Affiliations:** ^1^Department of Biology and Molecular Biosciences Program, Middle Tennessee State University, Murfreesboro, TN, United States; ^2^Computational Science Program, Middle Tennessee State University, Murfreesboro, TN, United States; ^3^Department of Biostatistics, University of Alabama at Birmingham, Birmingham, AL, United States; ^4^Department of Animal and Dairy Science, University of Georgia, Athens, GA, United States; ^5^National Center for Cool and Cold Water Aquaculture, USDA Agricultural Research Service, Kearneysville, WV, United States; ^6^Division of Animal and Nutritional Science, West Virginia University, Morgantown, WV, United States

**Keywords:** GWAS, SNP-chip, muscle, trout, fillet yield

## Abstract

Detection of coding/functional SNPs that change the biological function of a gene may lead to identification of putative causative alleles within QTL regions and discovery of genetic markers with large effects on phenotypes. This study has two-fold objectives, first to develop, and validate a 50K transcribed gene SNP-chip using RNA-Seq data. To achieve this objective, two bioinformatics pipelines, GATK and SAMtools, were used to identify ~21K transcribed SNPs with allelic imbalances associated with important aquaculture production traits including body weight, muscle yield, muscle fat content, shear force, and whiteness in addition to resistance/susceptibility to bacterial cold-water disease (BCWD). SNPs ere identified from pooled RNA-Seq data collected from ~620 fish, representing 98 families from growth- and 54 families from BCWD-selected lines with divergent phenotypes. In addition, ~29K transcribed SNPs without allelic-imbalances were strategically added to build a 50K Affymetrix SNP-chip. SNPs selected included two SNPs per gene from 14K genes and ~5K non-synonymous SNPs. The SNP-chip was used to genotype 1728 fish. The average SNP calling-rate for samples passing quality control (QC; 1,641 fish) was ≥ 98.5%. The second objective of this study was to test the feasibility of using the new SNP-chip in GWA (Genome-wide association) analysis to identify QTL explaining muscle yield variance. GWA study on 878 fish (representing 197 families from 2 consecutive generations) with muscle yield phenotypes and genotyped for 35K polymorphic markers (passing QC) identified several QTL regions explaining together up to 28.40% of the additive genetic variance for muscle yield in this rainbow trout population. The most significant QTLs were on chromosomes 14 and 16 with 12.71 and 10.49% of the genetic variance, respectively. Many of the annotated genes in the QTL regions were previously reported as important regulators of muscle development and cell signaling. No major QTLs were identified in a previous GWA study using a 57K genomic SNP chip on the same fish population. These results indicate improved detection power of the transcribed gene SNP-chip in the target trait and population, allowing identification of large-effect QTLs for important traits in rainbow trout.

## Introduction

Aquaculture provides sustainable production of food fish with high protein/low-saturated fat to satisfy increasing U.S. and worldwide demand. To enable increased production by the aquaculture industry and to meet the ever-growing demand for fish, we need fast/efficient growth and high-quality filets. However, a major constraint to increasing production efficiency is the lack of genetically improved strains of fish for aquaculture (Gjerde, 2006; World Fish Center, 2009). Development of tools that will enable genomic selection for improved aquaculture production traits will greatly benefit the aquaculture industry.

Fast/efficient muscle growth is a major trait affecting profitability of the aquatic muscle food industry. The genetic basis of muscle growth traits is not well studied in fish. Understanding molecular mechanisms of fish muscle growth can facilitate broodstock selection decisions. Skeletal muscle is the most abundant tissue and edible portion of fish and typically constitutes about 50–60% of the fish weight (Salem et al., 2006). Growth, development and quality traits of muscle are governed by organized expression of genes encoding contractile and regulatory proteins (Gerrard and Grant, 2003).

Genetic maps, characterizing the inheritance patterns of traits, and markers have been developed and used for a wide range of species, including fish. These tools target the discovery of allelic variation affecting traits with an ultimate goal of identifying DNA sequences underlying phenotypes (Rexroad et al., 2008). Markers have been identified with a variety of molecular techniques. Single nucleotide polymorphisms (SNPs) are abundant and distributed genome-wide, therefore, they are most suitable for high-throughput association studies (Wang et al., 2008; Gonzalez-Pena et al., 2016). Marker-assisted selection (MAS) can be used to improve breeding for phenotypes with large-effect QTLs. This method has been recently applied for the trait of infectious pancreatic necrosis virus (IPNV) resistance in Atlantic salmon (Houston et al., 2008; Moen et al., 2009). Genomic selection (GS) tools have been developed to increase the efficiency of genetic improvement in livestock compared to conventional pedigree-based selective breeding methods (Taylor et al., 2016). This concept has been recently demonstrated for bacterial cold-water disease (BCWD) resistance in rainbow trout aquaculture (Vallejo et al., 2017a). SNPs located within or near coding sequences, cSNPs, are especially important because they have the potential to change protein function (Brookes, 2007; Salem et al., 2012; Al Tobasei et al., 2017b). Therefore, cSNPs are particularly useful as genetic markers with large-effect on phenotypes, allowing MAS and improved accuracy of whole-genome selection. Because the muscle yield trait targeted in this study requires lethal sampling to measure the phenotype, only family-specific EBVs are available for breeding candidates in traditional breeding programs. The ability to use genomic selection or MAS will allow further within-family selection for the muscle yield trait, and thus is anticipated to increase the accuracy of genetic predictions and selection response.

Recently, we used an RNA-Seq approach to identify putative SNPs with allelic imbalances associated with total body weight, muscle yield, muscle fat content, shear force, and whiteness (Salem et al., 2012; Al Tobasei et al., 2017b). Similarly, RNA-Seq data were used to identify SNPs with allelic imbalances in fish families showing variations in resistance to *Flavobacterium psychrophilum*, the etiological agent of BCWD in rainbow trout (Marancik et al., 2014; Al Tobasei et al., 2017a). Together about 50 and 229K transcribed SNPs were identified in the two studies, respectively. Of them, ~21K SNPs had allelic-imbalances in families with contrasting phenotypes. The first objective of this study was to design, develop, and validate a 50K transcribed gene SNP-chip. The chip content includes the 21K transcribed SNPs with allelic-imbalances associated with the aforementioned traits and ~29K SNPs without allelic-imbalances that were strategically added to achieve more even genome-wide distribution. The new SNP-Chip is available from Affymetrix. The second objective of this study was to test the feasibility of using the new SNP-chip in GWA analysis to identify QTL explaining muscle yield variance in the USDA/NCCCWA rainbow trout growth-selected line. The results were compared with a previous GWA study for the same trait in the same population that we have previously conducted with a genomic-based 57K SNP chip (Gonzalez-Pena et al., 2016).

## Materials and methods

### Ethics statement

Institutional Animal Care and Use Committee of the United States Department of Agriculture, National Center for Cool and Cold Water Aquaculture (Leetown, WV) specifically reviewed and approved all husbandry practices and experimental procedures used in this study (Protocols #056 and 076).

### Source and selection of SNPs for the chip

Recently, we used RNA-Seq and two bioinformatic pipelines, GATK and SAMtools, for discovering coding/functional SNPs from 98 rainbow trout fish families (5 fish each) showing variations in whole-body weight, muscle yield, muscle fat content, shear force, and whiteness (Al Tobasei et al., 2017b). GATK detected 59,112 putative SNPs and SAMtools detected 87,066 putative SNPs. The two datasets contained approximately 50K non-redundant common SNPs; of which, 30,529 mapped to protein-coding genes (with 7.7% non-synonymous SNPs) and 4,386 mapped to lncRNAs. A total of 7,930 non-redundant SNPs had allelic imbalances between the low- and high-ranked families for the phenotypes. Validation of a subset of 92 SNPs revealed (1) 86.7–93.8% success rate in identifying polymorphic SNPs and (2) 95.4% consistent matching between DNA and cDNA genotypes, indicating a high rate of identifying SNPs using RNA-Seq. This SNP data set was recently published, Al Tobasei et al. (2017b) and is available through the NCBI dbSNP database (accession numbers ss#2711191806-2711287038 in addition to ss#2137497773).

Similarly, we identified transcribed gene SNPs in two genetic lines, ARS-Fp-R (resistant) and ARS-FP-S (susceptible), that were created by selective breeding to exhibit divergent resistance to BCWD. RNA-Seq analysis of pooled RNA samples was used to identify SNPs from the resistant and susceptible genetic lines. Fish belonging to resistant and susceptible genetic lines were collected on day 1 and day 5 post-challenge with Fp versus PBS injection (Marancik et al., 2014; Al Tobasei et al., 2017a). Using GATK bioinformatics pipelines, ~229K transcribed SNPs were identified(Al Tobasei et al., 2017a). The total number of SNPs with allelic imbalance, after removing redundant SNPs, was 7,951.

The SNPs identified in the previous two studies were used as a source to build the SNP array described in this study. About 21K transcribed SNPs with allelic-imbalances associated with the above-listed traits were included in the chip. These SNPs were identified from pooled RNA-Seq data collected from ~620 fish, representing 98 families from the ARS growth-selected line and 54 families from the ARS-Fp-R and -S lines. In addition, about 29K transcribed SNPs without allelic-imbalances were selected from all the putative SNPs and were strategically added to the chip with the aim of achieving even distribution of SNPs along the rainbow trout 29 chromosomes. The additional SNPs were selected to represent as many genes as possible in the genome: two SNPs were selected per gene from 14K genes with available SNPs. The chip includes ~5K non-synonymous SNPs. The chip has probe sets for a total number of 50,006 SNPs.

### Chip genotyping quality assessment

The SNP-chip was used in genotyping 1,728 fish from the USDA-ARS genetic lines. The Affymetrix SNPolisher software was used to calculate the chip SNP- and sample-metrics and assess QCs and filter samples/genotypes at the default setting (Palti et al., 2015). Forty-seven SNPs previously genotyped by a Fluidigm PCR-based assay (Al Tobasei et al., 2017b) were used to check quality of Affymetrix chip genotyping using 120 samples genotyped by both the chip and Fluidigm SNP assays. In addition, we confirmed the quality of the SNPs and the order of the samples included in the genotyping panel through pedigree check. Among the fish genotyped we included previously confirmed parental-pairs of nine families with 470 offspring and confirmed an average of 99.4% matching between offspring SNP genotypes and the genotypes of the expected parents.

### SNP genomic distribution and annotation

SNPs used in building the chip were identified using the first draft of the rainbow trout reference genome (Al Tobasei et al., 2017b). To update genomic coordinates according to the newly released genome assembly (GenBank assembly Accession GCA_002163495, RefSeq assembly accession GCF_002163495; Gao et al., 2018), SNPs were mapped by BLASTing the SNP probe sequences (70 nt) to the new genome sequence. Sequences with 100% identity match and no gap with single hits were assigned to the new genome position. Sequences with multiple hits were re-Blasted using probe size of 150 nt by adding 40 nt flanking sequence in both direction. A total of 45K SNPs out of 50K SNPs were successfully assigned to the new genome and were used for the GWA analyses.

SNPeff program was used to classify and annotate functional effects of the SNPs (Cingolani et al., 2012). The gff file of the new rainbow trout genome reference was used to determine position of the SNPs in a gene i.e. located within mRNA start and end positions (genic), within a CDS, 5′UTR or 3′UTR. SNPs not within start and end positions of mRNA were considered intergenic. Upstream/downstream intergenic SNPs were determined if located within 5 Kb of an mRNA. SNPs within lncRNAs were determined using gtf file of our previously reported lncRNA reference (Al-Tobasei et al., 2016). SNP annotation was performed by intersecting the SNPs bed file with the gff/gtf file using Bedtools software (Quinlan and Hall, 2010).

### Rainbow trout population and phenotypes used for GWA

Genome-wide association analysis was carried out using fish from a growth-selected line that has been previously described (Leeds et al., 2016). Briefly, this synthetic line is a 2-yr-old winter/spring-spawning population that was developed beginning in 2002, became a closed population in 2004, and since then has gone through 5 generations of genetic selection for improved growth performance. Fish from two consecutive generations (i.e., the third and fourth generations of growth selection) were included in this study. Phenotypic data and DNA samples were collected from 878 fish (406 fish representing 98 families from year-class (YC) 2010 and 472 fish representing 99 families from YC 2012). Among the 878 fish genotyped for GWAS, 40 fish were previously used for the discovery of the muscle yield associated SNP as described above (Al Tobasei et al., 2017b). The aforementioned SNP array was used for GWAS. Methods used to sample fish from each nucleus family and to characterize muscle yield have been described previously (Gonzalez-Pena et al., 2016). Eggs were hatched in spring water at 7–13°C to synchronize hatch times. Each family was stocked separately in 200-L tanks and hand-fed a commercial fishmeal-based diet beginning at swim-up. Neomales were developed from a subset of alevins from the previous year class by feeding 2 mg/kg of 17α-methyltestosterone for 60 d post-swim-up, and the masculinized females were used as sires for the following generation. At 5-months old, fish were uniquely tagged by inserting a passive integrated transponder, and tagged fish were combined and reared in 1,000-L communal tanks. Fish were fed a commercial fishmeal-based diet using automatic feeders. EBV were computed based on a two-trait model, 10-mo BW and thermal growth coefficient (TGC), using MTDFREML (Boldman et al., 1995). Each generation, EBV was used as selection criterion and mating decisions were made to maximize genetic gain while constraining the inbreeding rate to ≤1% per generation using EVA evolutionary algorithm (Berg et al., 2006). Data from masculinized fish were not used in the growth analysis.

Fish were harvested between 410 and 437 days post-hatch (mean body weight = 985 g; *SD* = 239 g), between 446 and 481 days post-hatch (mean body weight = 1803 g; SD = 305 g), for the 2010, and 2012 hatch years, respectively. Individual body weight data were recorded at harvesting. Fish were taken off feed 5 days before harvesting. For measurement of muscle yield when harvested at each of five consecutive weeks, approximately 100 fish (i.e., 1 fish per full-sib family per week) were anesthetized in approximately 100 mg/L of tricaine methane sulfonate (Tricaine-S, Western Chemical, Ferndale, WA) slaughtered, and eviscerated. Head-on gutted carcasses were packed in ice, transported to the West Virginia University Muscle Foods Processing Laboratory (Morgantown, WV), and stored overnight. The next day, carcasses were manually processed into trimmed, skinless filets by a trained faculty member and weighed; muscle yield was calculated as a percent of total body weight (Salem et al., 2013). The fish used in GWA had an average muscle yield of 48.91% (*SD* = 2.42).

### GWA analyses

Weighted single-step GBLUP (WssGBLUP) was used to perform GWA analysis as implemented in previous studies (Wang et al., 2012, 2014; Misztal et al., 2014). In addition to phenotypic data, wssGBLUP integrates genotype and pedigree information to increase estimation precision and detection power (Wang et al., 2012) in a combined analysis that is executed by the BLUPF90 software (Misztal et al., 2002).

The following mixed model was used for single trait analysis:

$$\begin{array}{l} y=Xb+Z_{1}a+Z_{2}w+e \end{array}$$

where *y* is the vector of the phenotypes, *b* is the vector of fixed effects including harvest group and hatch year, *a* is the vector of additive direct genetic effects (i.e., animal effect), *w* is the vector of random family effect, and *e* is the residual error. The matrices X, Z_1_, and Z_2_ are incidence matrices for the effects contained in *b, a*, and *w*, respectively. The additive direct genetic effect is a correlated effect with covariance structure given by ${H\sigma }_{a}^{2}$, where $\sigma_{a}^{2}$ is the additive direct genetic variance and **H** is the realized relationship matrix that combines pedigree and genomic relationships (Legarra et al., 2009). In the WssGBLUP mixed model equations, the inverse of **H** is used (Aguilar et al., 2010).

$$H^{-1}=A^{-1}+\left[\begin{array}{c} \text{​​​​​​​​​​​}0\;0\\ 0\;G^{-1}\;A_{22}^{-1} \end{array}\right]$$

where *A*^−1^ is the inverse of the pedigree relationship matrix and has the dimension of the number of animals in the pedigree; $A_{22}^{-1}$ is the inverse of the pedigree relationship matrix among genotyped animals and *G*^−1^ is the inverse of the genomic relationship matrix; both *G*^−1^ and $A_{22}^{-1}$ have the dimension of the number of genotyped animals. The random family effect is uncorrelated and just accounts for the fact the animals within the same family were raised in a common environment, and the covariance structure is given by ${I\sigma }_{w}^{2}$, where **I** is an identity matrix and $\sigma_{w}^{2}$ is the family variance.

As BLUP-based models consider the variance components are known, AIREMLF90 (Misztal et al., 2002) was used to estimate variance components for the additive direct genetic effect, random family effect, and residuals. Inbreeding was considered in all analyses, and was calculated using INBUPGF90 (Misztal et al., 2002) on 63,808 fish that represent five generations in the NCCCWA population. Quality control (QC) of genomic data was performed using BLUBF90 (Misztal et al., 2002) with the following parameters: SNP with minimum Allele Frequency (MAF) >0.05, SNP with call rate >0.90, animals with call rate >0.90, and SNP with a difference between observed and expected allele frequency < 0.15 (i.e., HWE test) were kept in the data. Out of a total of 50,006 SNPs, 35,322 SNPs passed QC.

In WssGBLUP, the weights for each SNP was assigned the same weight (e.g., 1.0, i.e., standard ssGBLUP) for the first iteration. For the second iteration, weights were calculated based on the SNP effects (û) estimated in the previous iteration as û^2^2*p*(1−*p*), where p is the current allele frequency. Each iteration was performed using three steps as follows: first, weight was assigned as described above; second, BLUPF90 (Misztal et al., 2002) was used to compute genomic estimated breeding values (GEBV) based on a realized relationship matrix (**H**) that combines pedigree (**A**) and genomic relationship matrix (**G**), the last considered weights for SNP; and third, postGSF90 (Misztal et al., 2002) was used to calculate SNP effects and weights based on sliding variance windows of 50 adjacent SNP. A total of 2 iterations were used. A window based on physical size (i.e., specific number of nucleotides) was not used to avoid biases due to uneven distributed SNPs in the new SNP chip. A Manhattan plot based on the proportion of additive genetic variance explained by the windows was created using the qqman package in R (Turner, 2014); the genomic windows explaining significant proportion of the additive genetic variance for muscle yield could be detected.

### Citrate synthase (CS) activity assay

GWA analysis (described below) showed a SNP window contained the CS gene associated with the genetic variance in muscle yield. To assess the potential effect of the SNPs in this gene, we measured the CS activity in 100 fish from the 2012 year-class as previously described (Brijs et al., 2017; Seite et al., 2018). Frozen muscle tissue samples were homogenized using electric homogenizer on ice followed by centrifugation at 1,000g for 15 min at 4°C. The supernatant was used to assess the total protein concentration and CS activity. Total protein concentration was assessed using a BCA protein assay kit at 562 nm with bovine serum albumin (BSA) as the standard. CS activity was determined from the rate of appearance of reduced DTNB (5,5′-dithiobis [2-nitrobenzoic]), which was monitored with a spectrophotometer at 412 nm (Ekstrom et al., 2017). For the CS assay, 10 μL of diluted tissue homogenate (1.0 mg/ ml) was incubated with 140 μL reaction medium (0.1 mM DTNB, 0.2 mM AcetylCoA, 0.15 mM oxaloacetic acid, pH 8.0). The absorbance was read in triplicate at 412 nm (25°C) after 4 min. CS activity was expressed as ΔOD/ mg protein.

## Results and discussion

### Chip genotyping quality assessment

The SNP-chip was used to genotype 1,728 fish. Out of 50,006 SNPs, 32,273 SNPs (64.5%) were characterized as high quality and polymorphic and 3,458 SNPs (6.9%) were high quality monomorphic (Table 1).

**Table 1:** SNP chip Metric summary.

**Conversion Type**	**Count**	**Percentage**
Poly High Resolution	32,273	64.5
Other	8,395	16.7
Mono High Resolution	3,458	6.9
No Minor Hom	2,725	5.4
Call Rate Below Threshold	2,705	5.4
Off target variant	450	0.9

The Affymetrix SNPolisher software was used to filter samples/genotypes at the default setting (Palti et al., 2015). Out of 1,728 genotyped samples, 1,641 (94.9%) fish samples were retained, and 87 samples were filtered out because they failed to meet the 0.97 call rate (CR) and 0.82 Dish QC (DQC) thresholds. The average QC call rate for the passing samples was 99.6% (Table 2).

**Table 2:** SNP chip Sample QC Summary.

Number of input samples	1,728
Samples passing DQC	1,722
Samples passing DQC and QC CR	1,641
Samples passing DQC, QC CR and Plate QC	1,641 (94.9%)
Number of failing samples	87
Number of Samples Genotyped	1,641
Average QC CR for the passing samples	99.66

We compared the Affymetrix genotyping results of 47 SNPs that were previously genotyped by a Fluidigm PCR-based assay (Al Tobasei et al., 2017b). Using 120 samples genotyped by both methods, there was a 99.5% match in genotypes between the two assays for high-resolution polymorphic markers (data not shown). This test demonstrates the high quality of the SNP chip and reliable genotyping data for the subsequent GWA analyses.

**Figure 1:**
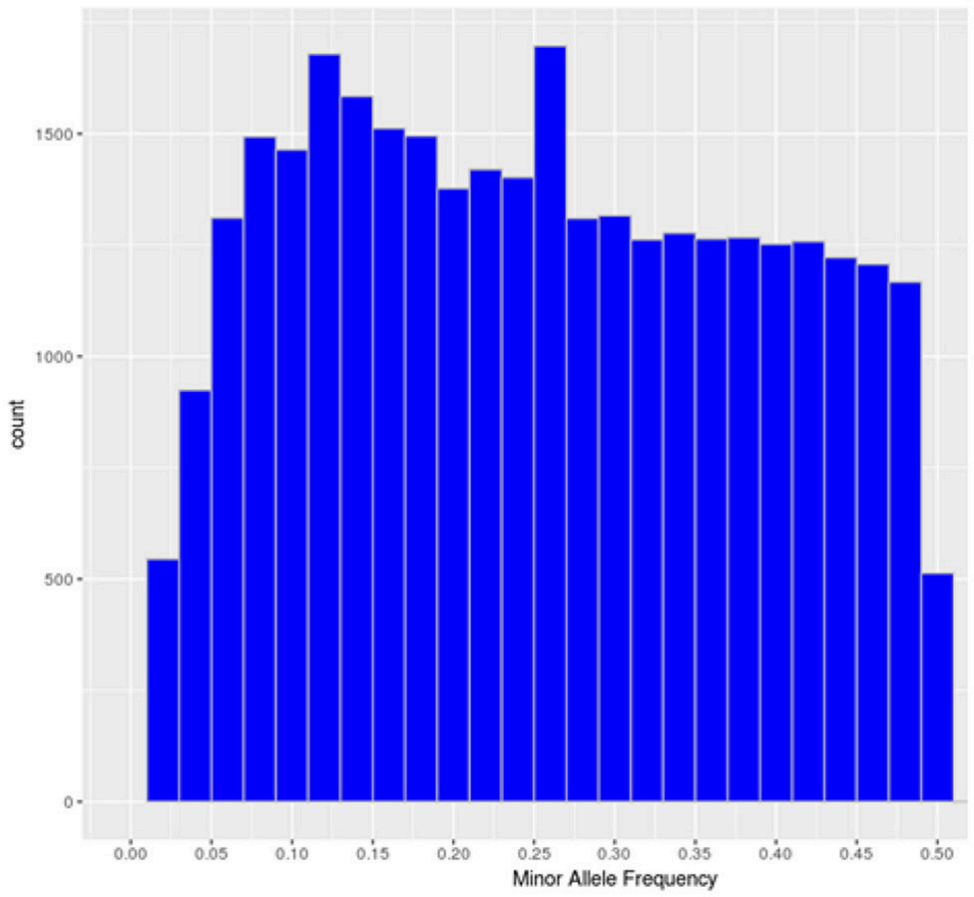
Minor allele frequency distribution of the polymorphic high-resolution SNPs in the SNP chip.

The SNP-chip showed an average minor allele frequency (MAF) of 0.25 and standard deviation of 0.134. A total of 27,280 SNPs had MAF> 0.1 and 16,101 SNP more than 0.25 (Figure 1).

### SNP density and genomic distribution

**Figure 2:**
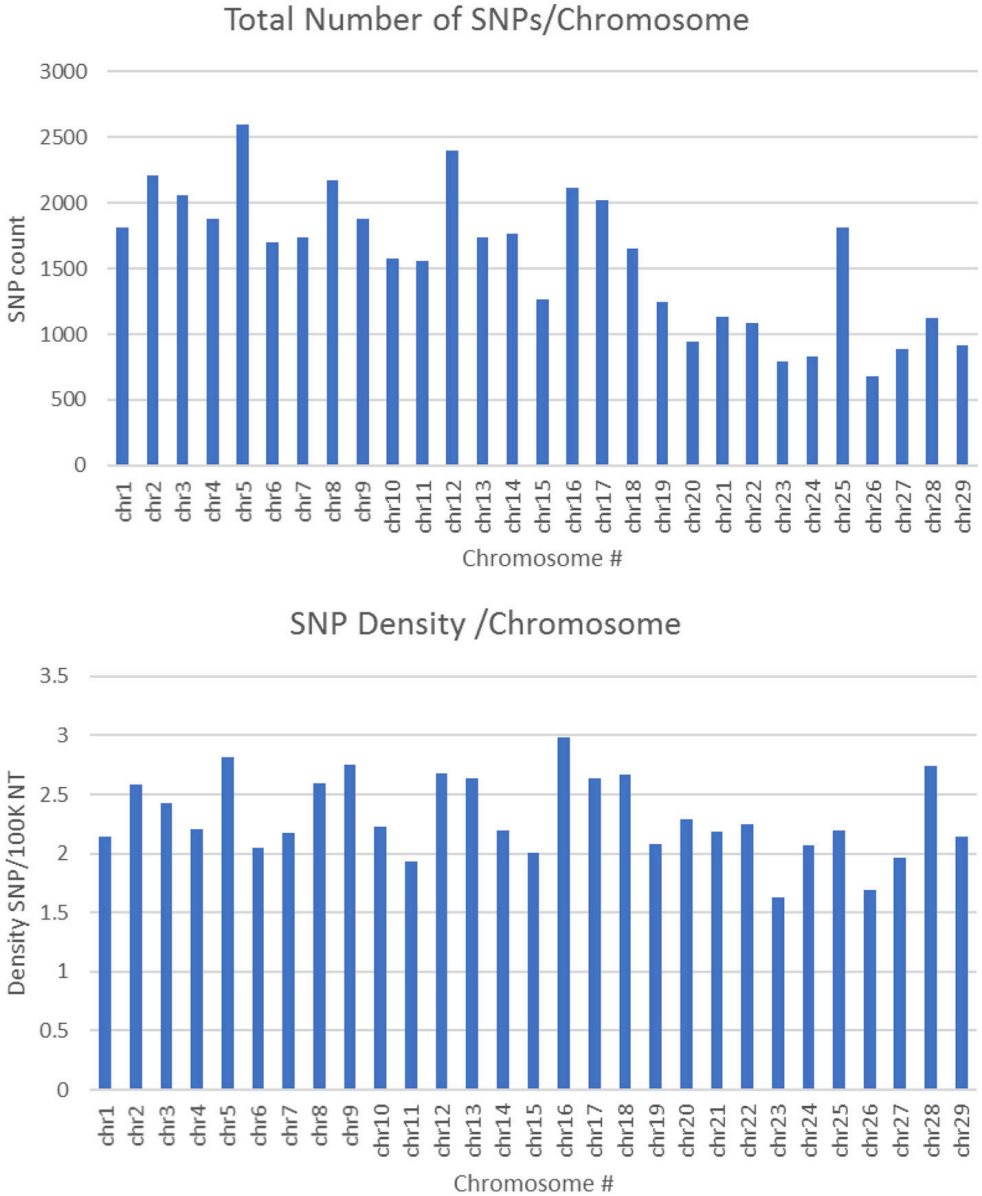
Number of SNPs per chromosome and SNP density distribution (SNP/100K nucleotide).

SNPs used in this study to build the chip were initially identified using a rainbow trout reference genome published by Berthelot et al. in 2014 (Berthelot et al., 2014). However, in this reference only ~1 Gb out of a 2.1 Gb total length of the assembly is anchored to chromosomes. Recently, a newer genome assembly has been built that is currently available at NCBI (Accession GCA_002163495) (Gao et al., 2018). The new assembly has a 1.94 Gb total length (89% of the genome) anchored to 29 chromosomes. A total of 45,590 SNPs out of 50,006 existing in the SNP-chip were mapped to the new genome assembly with an average of 1,572 SNPs per chromosome. The average SNP density was 1 SNP per 42.7 Kb, with a range of 1 SNP/33.5 Kb (Chromosome 16) to 1 SNP/61.6 Kb (Chromosome 23). Figure 2 shows the number of SNPs per chromosome and the SNP density distribution. A total of 21K out 50K SNPs on the chip were selected based on putative association with phenotypic traits, and hence, were expected to be clustered in specific genome loci. However, supplementing the chip with 29K SNPs (two SNPs per gene) perhaps helped in randomizing the SNP distribution in the genome. Previously, a 57K genome-wide SNP array for rainbow trout reported an average of 1,551.4 mapped SNPs per chromosome (Gonzalez-Pena et al., 2016). The 57K array was designed primarily using SNPs originating from RAD-Seq sequencing of doubled-haploid clonal lines (Palti et al., 2014) and whole genome re-sequencing of fish from the Aquagen (Norway) breeding program. A key point here, is that the SNPs included in the 57K chip were originated from other genetic lines. Hence, although polymorphic enough in the NCCCWA growth line used in this study for conducting GWA as we have previously shown (Gonzalez-Pena et al., 2016), the SNPs used for GWA in this study were originated from the investigated population and were expected to be more informative due to ascertainment bias (Vallejo et al., 2017b).

### SNP annotation and classification based on functional effects

**Figure 3:**
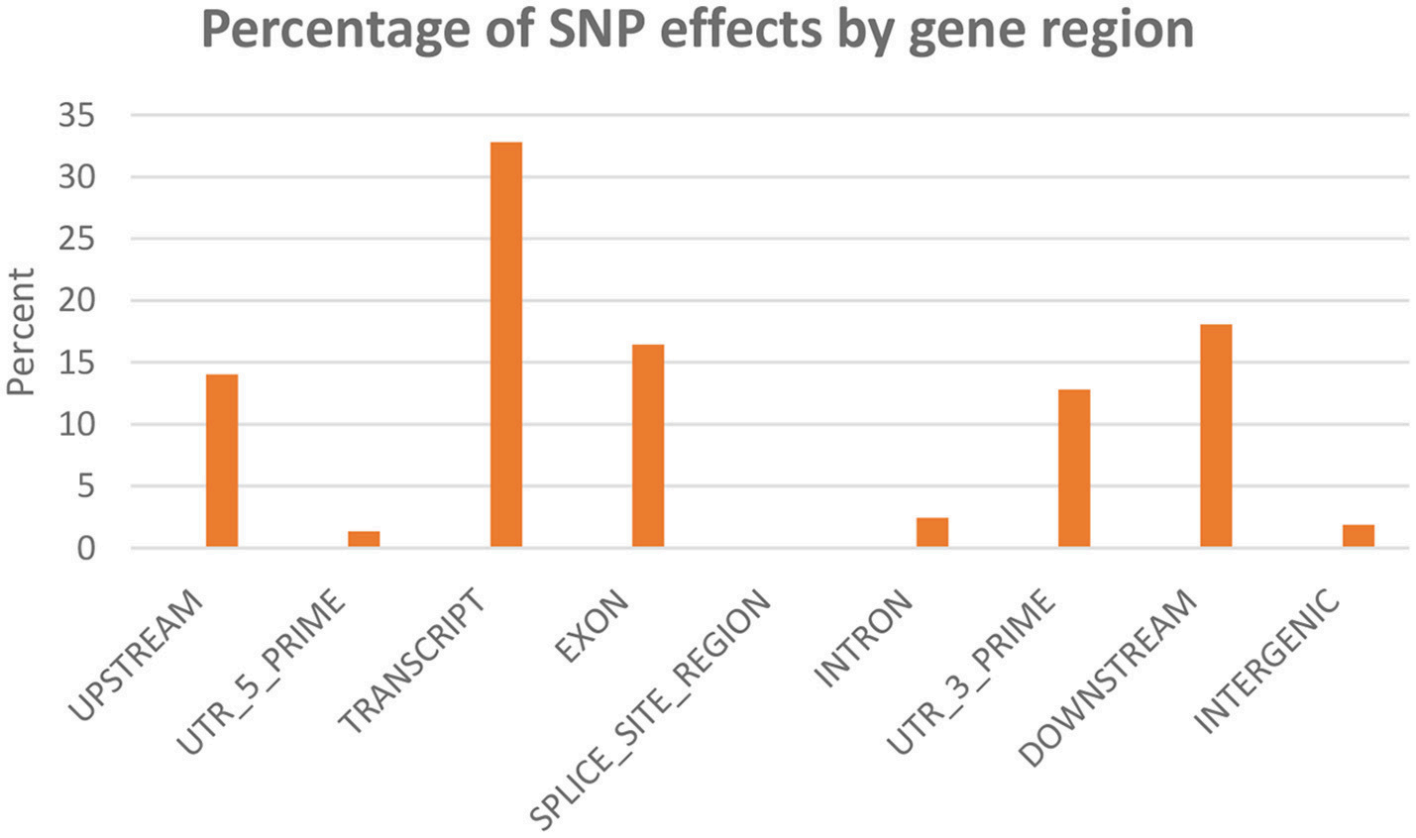
Percentage of SNP effects by gene region.

SNPeff program was used to classify and annotate functional effects of the SNPs. A total of 45,590 SNPs were included in this analysis. Classifying SNPs by impact showed 636 effects (0.23%) with high impact (stop-gain) and 20,987 effects (7.86%) with moderate impact, (missense variants). The rest (91.9%) represents low to moderate variant effect including synonymous and non-coding SNPs. Figure 3 shows percent of SNP effects by gene regions. A total of 32.8% of the effects were within transcripts with 16.5% exonic, 1.3% in the 5′-UTR and 12.8% in 3′-UTR. All SNPs on the chip were identified through transcriptome sequencing. Surprisingly, there were 14% upstream and 18.1% downstream effects (within 5 Kb of the genes). The upstream/downstream percent is consistent with our previous report that showed 17.1–20.2% SNPs within 5 Kb upstream/downstream of protein-coding genes in one of two SNP data sets used in building the SNP-chip (Al Tobasei et al., 2017b). On the other hand, there was only 1.9% of the SNP effects within intergenic regions, compared to 37.7–49.2% intergenic SNPs in the previous study (Al Tobasei et al., 2017b). In our previous study, the high percentage of intergenic and upstream/downstream SNPs was explained by the incomplete annotation of protein-coding genes and exons used in the previous version of the rainbow trout reference genome (Berthelot et al., 2014). The drop in the percentage of intergenic SNP effects in this study may be due to the improved gene annotation of the current version of the genome reference.

### GWA analyses

#### Genomic regions associated with muscle yield

**Figure 4:**
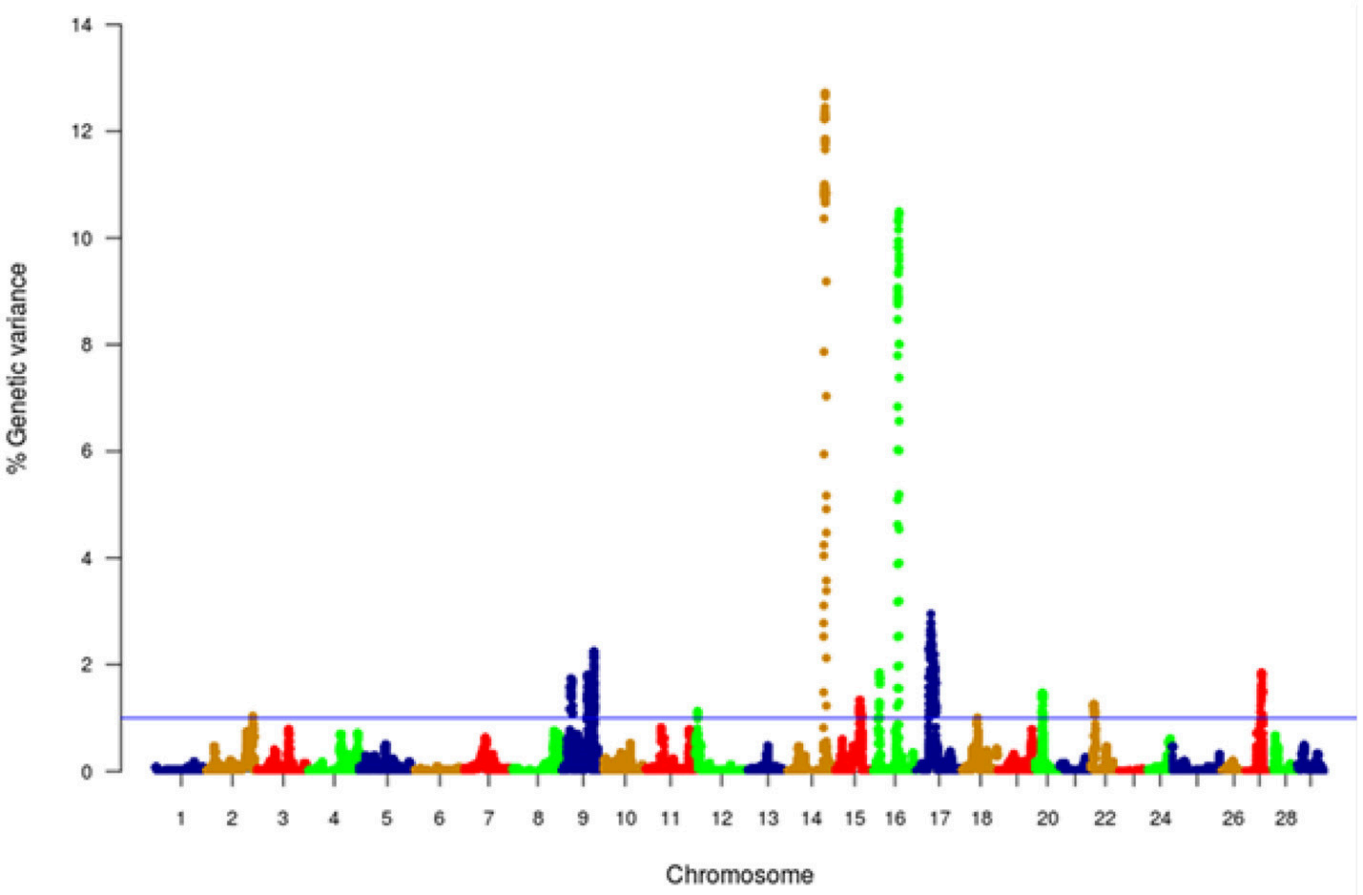
Manhattan plot of GWA analysis performed with WssGBLUP and showing association between SNP genomic sliding windows of 50 SNPs and muscle yield. Chromosomes 14 and 16 showed the highest peaks with genomic loci, explaining together up to 23.2% of the genetic variance. The blue line shows a threshold of 1% of additive genetic variance explained by SNPs.

GWA analysis using WssGBLUP identified 163 SNPs, each explaining at least 2% of the genetic variance of muscle yield (Tables 3, 4; Supplementary File 1). The SNPs were clustered into 4 main chromosomes (14, 16, 9, and 17). Chromosomes 14 and 16 showed the highest peaks with genomic loci explaining up to 12.71% and 10.49% of the genetic variance, respectively. The total variance explained by these loci is 23.2%. Figure 4 shows a Manhattan plot displaying association between SNP genomic sliding window of 50 SNPs and muscle yield. Sixty-nine of the 163 SNPs (42.2%) were previously identified as SNPs with allelic imbalances associated with muscle yield in the original SNP data set used to build the SNP chip (Al Tobasei et al., 2017b). Twenty-one of the 163 SNPs caused non-synonymous mutations. The rest of the SNPs were either silent mutations or located in UTR of the genes indicating their potential epigenetic mechanism of gene regulation. Important SNPs with more than 5% genetic variance are discussed below and all 163 SNPs are listed in Supplementary File 1.

**Table 3:** Selected SNP markers explaining the largest proportion of genetic variance (>5%) for muscle yield in chromosome 14 using 50 adjacent SNP windows.

**Variance %**	**CHR**	**SNP position**	**Distance to next SNP**	**Strand**	**Gene**	**Annotation**	**Region/Effect**
5.95	14	60291342	16113	+	etfdh	Electron Transfer Flavoprotein Dehydrogenase	CDS/nonSyn
7.86	14	60307455	366	+	etfdh	Electron Transfer Flavoprotein Dehydrogenase	CDS/syn
10.36	14	60307821	8	+	etfdh	Electron Transfer Flavoprotein Dehydrogenase	3′UTR
10.79	14	60307829	2256	+	etfdh	Electron Transfer Flavoprotein Dehydrogenase	3′UTR
10.84	14	60310085	163538	-	ppid	Peptidylprolyl Isomerase D	CDS/nonSyn
10.90	14	60473623	421210	+	rapgef2	Rap Guanine Nucleotide Exchange Factor 2	3′UTR
10.96	14	60894833	295302	NA	NA	NA	NA
11.00	14	61190135	558	+	LOC110488945	Prominin-1-A	3′UTR
11.00	14	61190693	7552	+	LOC110488945	Prominin-1-A	3′UTR
2.24	14	61198245	76178	+	LOC110488947	Fibroblast Growth Factor-Binding Protein 1	CDS/nonSyn
12.23	14	61274423	13691	–	LOC110488948	Cyclin-A2	3′UTR
12.29	14	61288114	762	–	LOC110488950	Transmembrane Protein 33	3′UTR
12.35	14	61288876	528124	–	LOC110488950	Transmembrane Protein 33	CDS/syn
12.36	14	61817000	18067	–	LOC110488956	Protein Farnesyltransferase/Geranylgeranyltransferase Type-1 Subunit Alpha	3′UTR
12.30	14	61835067	6866	–	LOC110488957	Glutathione S-Transferase P	3′UTR
12.26	14	61841933	319532	–	LOC110488957	Glutathione S-Transferase P	CDS/syn
12.24	14	62161465	1101	NA	NA	NA	NA
12.45	14	62162566	79441	NA	NA	NA	NA
12.71	14	62242007	38699	–	LOC110488962	Inositol Polyphosphate 5-Phosphatase Ocrl-1	CDS/nonSyn
12.71	14	62280706	12616	+	LOC110488963	Chloride Intracellular Channel Protein 2	3′UTR
12.70	14	62293322	4394	+	LOC110488964	C1Galt1-Specific Chaperone 1	3′UTR
12.65	14	62297716	9021	–	mcts1	Mcts1, Re-Initiation And Release Factor	3′UTR
11.85	14	62306737	36808	–	mcts1	Mcts1, Re-Initiation And Release Factor	5′UTR
11.84	14	62343545	586	+	lamp2	Lysosomal Associated Membrane Protein 2	CDS/nonSyn
11.85	14	62344131	2211	+	lamp2	Lysosomal Associated Membrane Protein 2	CDS/nonSyn
11.78	14	62346342	306	+	lamp2	Lysosomal Associated Membrane Protein 2	Intronic
11.78	14	62346648	579	+	lamp2	Lysosomal Associated Membrane Protein 2	Intronic
11.77	14	62347227	29198	+	lamp2	Lysosomal Associated Membrane Protein 2	Intronic
11.66	14	62376425	304	+	tmem255a	Transmembrane Protein 255A	CDS/syn
10.92	14	62376729	3620	+	tmem255a	Transmembrane Protein 255A	CDS/syn
10.87	14	62380349	282	+	tmem255a	Transmembrane Protein 255A	3′UTR
10.86	14	62380631	31094	+	tmem255a	Transmembrane Protein 255A	3′UTR
10.86	14	62411725	1632	+	upf3b	Upf3B, Regulator Of Nonsense Mediated Mrna Decay	CDS/nonSyn
10.86	14	62413357	1931	+	upf3b	Upf3B, Regulator Of Nonsense Mediated Mrna Decay	3′UTR
10.92	14	62415288	26359	+	LOC110488974	60S Ribosomal Protein L39	3′UTR
10.90	14	62441647	10087	+	LOC110488975	Septin-6	CDS/syn
10.88	14	62451734	10231	+	LOC110488975	Septin-6	CDS/syn
10.88	14	62461965	6983	+	LOC110488975	Septin-6	3′UTR
10.75	14	62468948	89647	NA	NA	NA	NA
10.72	14	62558595	7052	+	LOC110488979	Ets-Related Transcription Factor Elf-1	3′UTR
10.66	14	62565647	66310	–	LOC110488980	Tenomodulin	3′UTR
10.67	14	62631957	1503911	–	LOC110488980	Tenomodulin	CDS/nonSyn
10.83	14	64135868	6948	+	gla	Galactosidase Alpha	CDS/nonSyn
9.18	14	64142816	2581	–	LOC110488986	60S Ribosomal Protein L36A	CDS/syn
7.03	14	64145397	20716	–	LOC110488986	60S Ribosomal Protein L36A	CDS/nonSyn
5.17	14	64166113		+	btk	Bruton Tyrosine Kinase	CDS/nonSyn

*Color intensities reflect changes in additive genetic variance (green is the highest and red is the lowest)*.

**Table 4:** Selected SNP markers explaining the largest proportion of genetic variance (>5%) for muscle yield in chromosome 16 using 50 adjacent SNP windows.

**Variance %**	**CHR**	**SNP position**	**Distance to next SNP**	**Strand**	**Gene**	**Annotation**	**Region/Effect**
4.62	16	39953311	12000	+	tnfrsf5a	Tnf Receptor Superfamily Member 5A Precursor	5'UTR
5.09	16	39965311	3	+	tnfrsf5a	Tnf Receptor Superfamily Member 5A Precursor	CDS/nonSyn
6.03	16	39965314	689	+	tnfrsf5a	Tnf Receptor Superfamily Member 5A Precursor	CDS/nonSyn
6.83	16	39966003	608	+	tnfrsf5a	Tnf Receptor Superfamily Member 5A Precursor	CDS/nonSyn
7.79	16	39966611	666	+	tnfrsf5a	Tnf Receptor Superfamily Member 5A Precursor	3'UTR
8.47	16	39967277	149527	+	tnfrsf5a	Tnf Receptor Superfamily Member 5A Precursor	3'UTR
8.76	16	40116804	438	NA	NA	NA	NA
9.06	16	40117242	5021	–	LOC110492067	Kelch Protein 21	CDS/syn
9.04	16	40122263	471	–	LOC110492067	Kelch Protein 21	CDS/syn
9.04	16	40122734	206269	–	LOC110492067	Kelch Protein 21	CDS/syn
8.97	16	40329003	423	+	LOC110492070	45 Kda Calcium-Binding Protein	3'UTR
8.88	16	40329426	430961	+	LOC110492070	45 Kda Calcium-Binding Protein	3'UTR
8.88	16	40760387	133719	+	LOC100136676	Caspase-9	CDS/syn
8.87	16	40894106	16043	+	LOC110491067	Basement Membrane-Specific Heparan Sulfate Proteoglycan Core Protein	CDS/syn
8.81	16	40910149	15660	+	LOC110491067	Basement Membrane-Specific Heparan Sulfate Proteoglycan Core Protein	CDS/nonSyn
8.81	16	40925809	134	NA	NA	NA	NA
8.82	16	40925943	328	NA	NA	NA	NA
8.88	16	40926271	36300	NA	NA	NA	NA
8.88	16	40962571	1603	+	LOC110492082	Cdp-Diacylglycerol–Serine O-Phosphatidyltransferase	3'UTR
8.88	16	40964174	1011	NA	NA	NA	NA
8.89	16	40965185	134	NA	NA	NA	NA
8.89	16	40965319	214946	NA	NA	NA	NA
9.33	16	41180265	15995	+	LOC110492084	Membrane-Associated Guanylate Kinase, Ww And Pdz Domain-Containing Protein 3	CDS/syn
9.37	16	41196260	49825	–	LOC110492085	Tyrosine-Protein Phosphatase Non-Receptor Type 12	CDS/syn
9.82	16	41246085	3112	+	LOC100136105	Complement Receptor	CDS/syn
9.82	16	41249197	474	+	LOC100136105	Complement Receptor	3'UTR
9.83	16	41249671	30475	+	LOC100136105	Complement Receptor	3'UTR
9.95	16	41280146	574	+	c4bp	C4B-Binding Protein Alpha Chain	3'UTR
9.93	16	41280720	774	+	c4bp	C4B-Binding Protein Alpha Chain	3'UTR
10.15	16	41281494	229	NA	NA	NA	NA
10.29	16	41281723	24001	NA	NA	NA	NA
10.33	16	41305724	20095	–	LOC110492088	Uncharacterized Loc110492088	NA
10.36	16	41325819	685099	–	cd34a	Cd34A Molecule	3'UTR
10.44	16	42010918	5137	–	slc26a9	Solute Carrier Family 26 Member 9	CDS/nonSyn
10.45	16	42016055	176696	–	slc26a9	Solute Carrier Family 26 Member 9	CDS/syn
10.49	16	42192751	41683	–	LOC110492098	Cysteine/Serine-Rich Nuclear Protein 2	CDS/syn
9.58	16	42234434	23274	+	LOC110492102	Daz-Associated Protein 2	3'UTR
9.68	16	42257708	1026	–	LOC110492103	Rac Gtpase-Activating Protein 1	3'UTR
9.45	16	42258734	38505	–	LOC110492103	Rac Gtpase-Activating Protein 1	3'UTR
8.01	16	42297239	2891	+	LOC110492108	Citrate Synthase, Mitochondrial	CDS/nonSyn
8.01	16	42300130	5927	+	LOC110492108	Citrate Synthase, Mitochondrial	CDS/nonSyn
7.38	16	42306057	101	+	LOC110492108	Citrate Synthase, Mitochondrial	3'UTR
6.57	16	42306158	92	+	LOC110492108	Citrate Synthase, Mitochondrial	3'UTR
6.01	16	42306250	1	+	LOC110492108	Citrate Synthase, Mitochondrial	3'UTR
5.19	16	42306251	60	+	LOC110492108	Citrate Synthase, Mitochondrial	3'UTR
4.54	16	42306311	303	+	LOC110492108	Citrate Synthase, Mitochondrial	3'UTR
3.90	16	42306614	605	+	LOC110492108	Citrate Synthase, Mitochondrial	3'UTR
3.19	16	42307219	57	+	LOC110492108	Citrate Synthase, Mitochondrial	3'UTR
2.53	16	42307276		+	LOC110492108	Citrate Synthase, Mitochondrial	3'UTR

*Color intensities reflect changes in additive genetic variance (green is the highest and red is the lowest)*.

With 46 SNPs clustered into 23 annotated genes, chromosome 14 had the most significant QTL windows explaining up to 12.71% of the genetic variance in muscle yield (Table 3 and Figure 4). At least four genes can be inferred to be involved in cell differentiation/proliferation and regulation of gene expression based on their RefSeq annotation. The list included fibroblast growth factor-binding protein-1(FGFBP1) which had a single nonsynonymous SNP found in a window that explained 12.24% of the additive genetic variance. FGFBP1 plays an essential role in cell proliferation and differentiation by binding to fibroblast growth factors. The FGFBP1 expression increases during development and decreases before neuromuscular junction degeneration during aging (Taetzsch et al., 2017). The list of genes on chromosome 14 also includes inositol polyphosphate 5-phosphatase (OCRL-1). OCRL is involved in terminating the PI3K signaling and thus plays an important role in modulating effects of growth factors and insulin stimulation in cell proliferation and survival (Ooms et al., 2009). Prominin-1-A gene (PROM1) that encodes for a transmembrane glycoprotein had 2 SNPs. PROM1, often used as adult stem cell marker, plays a role in maintaining stem cell properties by suppressing differentiation (GeneCards-Human-Gene-Database, 2018a). Another gene on chromosome 14 was farnesyltransferase/geranylgeranyltransferase type-1 subunit alpha (FNTA) which had a SNP explaining 12.36% of the variance. FNTA may positively regulate neuromuscular junction development (UniProtKB, 2018a).

In addition, chromosome 14 had three genes involved in the cell cycle regulation. The first gene is MCTS1 re-initiation and release factor that had two SNPs in a window explaining 12.65% of the additive genetic variance. MCTS1 is anti-oncogene that decreases cell doubling time by shortening the G1 and G1/S transit time (GeneCards-Human-Gene-Database, 2018b). The second cell cycle control gene was cyclin-A2 which promotes transition through G1/S and G2/M and can block muscle-specific gene expression during muscle differentiation (Skapek et al., 1996). The third gene was glutathione S-transferase P (GSTP1). Although involved in numerous biological functions, GSTP1 negatively regulates CDK5 activity via p25/p35 translocation which diminishes neurodegeneration (Sun et al., 2011).

Chromosome 14 also had SNPs in genes playing important mitochondrial functions. There were 4 SNPs in the gene encoded for the electron transfer flavoprotein dehydrogenase (ETFDH) which is an important enzyme in the mitochondrial electron transport chain. Mutations in ETFDH are associated with myopathies (Haller and DiMauro, 2012). Another mitochondrial-relevant gene was peptidylprolyl isomerase D (PPID). Mutations in PPID are associated with muscular dystrophy in human (Giorgio et al., 2010).

Few other genes included in the QTL region on chromosome 14 are important for maintenance of the muscle functions. Of them is the chloride intracellular channel protein 2 (CLIC2) which modulates the activity of ryanodine receptor 2 (RYR2) and inhibits calcium influx, and therefore is involved in regulating muscle contraction (Ekstrom et al., 2017). Five SNPs were in the lysosomal-associated membrane protein 2 gene (LAMP2). LAMP2 mutations were reported in patients with cardioskeletal myopathies (Berthelot et al., 2014). Two SNPs were located in the UPF3B gene, a regulator of nonsense-mediated mRNA decay (NMD). NMD inhibition was observed in patients with muscular dystrophy (Palti et al., 2014). Three SNPs were observed in the septin-6 gene. Mutations of septin-9 (another gene family member) is genetically linked to muscle atrophy (Vallejo et al., 2017b). Two SNPs were identified in the tenomodulin gene which showed downregulation in an animal muscle atrophy model (Taetzsch et al., 2017).

Chromosome 16 ranked second in having the most significant QTL windows with 49 SNPs clustered into 16 annotated genes (Table 4 and Figure 4). The gene within the most significant SNP window to additive genetic variance was the cysteine/serine-rich nuclear protein 2 (CSRNP2). CSRNP2 has DNA binding transcription factor/activation activity. Deletion of CSRNP1/2/3 three gene family members resulted in mice neonatal lethality (Gingras et al., 2007). Another gene within the same SNP window was solute carrier family 26 member 9 (Slc26a9). Little is known about the function of Slc26a9 in muscle, it serves as anion exchanger mediating chloride, sulfate and oxalate transport and chloride/bicarbonate exchange (UniProtKB, 2018b). A single SNP was observed in the stem cell marker CD34a gene. Cd34(^−/−^) mice showed a defect in muscle regeneration caused by acute or chronic muscle injury (Alfaro et al., 2011).

Several genes were involved in cell signaling/receptor activity. Five SNPs were predicted in 2 genes of the immune-related complement activation pathway, these are the complement receptor and C4b-binding protein alpha chain. Recent studies indicated that the complement is activated as a response of skeletal muscle injury and plays a key role during muscle regeneration (Zhang et al., 2017). A single SNP was identified in the tyrosine-protein phosphatase non-receptor type 12 (PTPN12) which dephosphorylates a wide-range of proteins, and thus regulates several cellular signaling cascades such as ERBB2 and PTK2B/PYK2 (UniProtKB, 2018c). This group of genes also includes the membrane-associated guanylate kinase, WW and PDZ domain-containing protein 3 (MAGI3), which is involved in the regulation of various cell signaling processes including the AKT1, TGFA, ERK and JNK signaling cascades (UniProtKB, 2018d). Two SNPs were in the basement membrane-specific heparan sulfate proteoglycan core protein (HSPG2). A mouse model deficient in this gene showed muscle hypertrophy through reduced myostatin expression suggesting a role in maintaining fast muscle mass and fiber composition (Xu et al., 2010). Five SNPs were in the TNF receptor superfamily member 5A gene. Recently, some proinflammatory cytokines belonging to TNF superfamily have been recognized as an important regulator of skeletal muscle mass (Tajrishi et al., 2014).

Chromosome 16 also had a single SNP in the DAZ-associated protein 2 (DAZAP2). Not much is known about the DAZAP2 function in muscle, however, DAZAP2 interacts with the transforming growth factor-beta signaling molecule SARA (Smad anchor for receptor activation), eukaryotic initiation factor 4G, and an E3 ubiquitinase (Giorgio et al., 2010). Another gene in the list was Rac GTPase-activating protein 1 (RACGAP1) that harbored 2 SNPs explaining up to 9.658% of the genetic variance. RACGAP1 regulates cytokinesis and cell differentiation (Wang et al., 2018). A single SNP existed in caspase-9 which has an important non-apoptotic role in muscle differentiation (Murray et al., 2008). Three SNPs were located in the kelch protein 21. Several Kelch family members play important roles in skeletal muscle development by regulating the cell proliferation and/or differentiation (Gupta and Beggs, 2014).

An important gene affecting muscle function which is also located within the QTL region on chromosome 16 is the citrate synthase (CS), which is used as a marker for human mitochondrial functions. Ten SNPs explaining up to 8% of the genetic variance were located in the CS gene.

GWA studies in fish to identify QTL affecting muscle yield and quality are still in its infancy. Previous GWA analysis using a 57K genomic SNP chip on the same fish population identified two windows that explained 1.5 and 1.0% of the additive genetic variance for muscle yield and 1.2 and 1.1% for muscle weight. Interestingly, the windows are located on chromosome 9, which showed some association with muscle yield in the current study; however, none of the SNPs were annotated to the same genes. No major QTLs were identified in the previous study. This large difference in the outcomes of the two studies was somewhat unexpected. However, it may be explained by lower marker density within or near genes in the 57K chip (Gonzalez-Pena et al., 2016) and by ascertainment bias, because the transcribed SNPs used in this study were discovered in the phenotyped fish and hence are expected to be more polymorphic and informative for GWA analysis in this population. Additionally, in this study, sliding windows of 50 SNP were used contrasting with 20 non-sliding windows in the previous study. Difference in window size slightly contributed to the increased proportion of variance (data not shown). By using SNP windows, it is assumed that those DNA blocks may be inherited together, which may not always be the case for all assumed windows. In common carp, genetic linkage mapping identified QTLs with large effects for muscle fiber cross-section area (21.9%) and muscle fiber density (18.9%) (Zhang et al., 2011). Genome-wide significant QTL affecting growth and muscle related traits were identified in Atlantic salmon (Tsai et al., 2015). The latter two studies, together with our study, indicate existence of large-effect QTLs affecting muscle yield in aquaculture species. However, the QTLs identified in this study might be population specific and thus, need to be tested in other populations.

#### Citrate synthetase activity correlation with muscle yield

**Figure 5:**
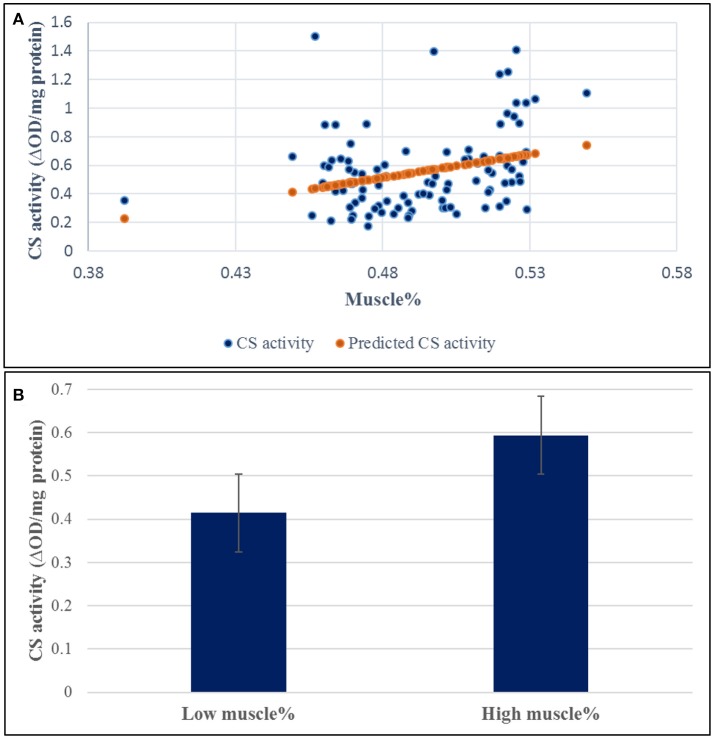
Correlation coefficient between muscle yield and CS activity in 96 samples. **(A)** The regression coefficient R2 value between the muscle yield and CS activity was 0.092 (*p*-value 0.002). **(B)** CS had 1.43-fold increase in the high-ranked fish compared to the low ranked ones.

A SNP window on chromosomes 16 explaining up to 8.01% of the genetic variance in muscle yield contained 10 SNPs of the CS gene. Two of the SNPs were nonsynonymous mutations. To investigate the potential effect of these SNPs, we measured the CS activity in 100 fish from the 2012 year-class. The samples included 38 fish from 5 high-ranked and 5 low-ranked families for muscle yield, 19 each, and 62 randomly selected fish. CS had 1.43-fold increase in the high-ranked fish compared to the low ranked ones (Figure 5). The regression coefficient R^2^ value between the muscle yield and CS activity was 0.092 (*p*-value 0.002). However, there was no significant association between any of the SNP genotypes and CS activity (*P*-value < 0.001). Mitochondria are at the center of age-related sarcopenia that is characterized by decline in human muscle mass. Skeletal muscle CS decreases with aging in humans (Short et al., 2005). Therefore, our results suggest an important role of mitochondrial functions to muscle growth.

### Conclusion

This study provides a 50K transcribed gene SNP-chip based on RNA-Seq data from fish families showing genetic diversity for six aquaculture production traits in the USDA/NCCCWA growth- and disease-selected genetic lines. The chip was tested for GWA analysis, which led to identification of large-effect QTL for muscle yield in that population. Other muscle quality traits are currently under investigation. Collectively, these studies will allow the use of SNP markers to estimate breeding values for muscle yield and quality traits that are economically important traits for aquatic food producers, processors, and consumers. Current and future selection at the NCCCWA will select for improved filet yield. Genetic markers are desirable for these traits because genetic improvement is limited by the inability to measure filet yield traits directly on broodstock due to lethal sampling. Hence the accuracy and efficiency of selective breeding can be improved by taking advantage of the genomic information, even though limited phenotyping is available for this economically-important trait.

One potential limitation in this study is the use of the same population for SNP identification and GWAS. The QTLs identified in this study might be population specific and thus, need to be tested in other populations. It is worth mentioning that while the SNP chip has 50K SNPs, about 7.9K SNPs had putative allelic imbalances associated with 5 growth and muscle related traits (body weight, muscle yield, muscle fat content, shear force, and whiteness). Also, there were 13K additional SNPs with putative allelic imbalances associated with resistance BCWD. About 620 fish were used in the previous RNA-Seq analyses to identify these putative SNPs (Al Tobasei et al., 2017a,b). In this study, only one of the 6 traits, muscle yield was considered for GWAS. Only 40 fish were used in the previous RNA-Seq study to identify the putative SNPs that were associated with muscle yield (Al Tobasei et al., 2017b). To make sure those fish do not affect the GWAS results, we removed those 40 fish in addition to all fish involved in determining the growth/muscle putative SNPs (a total of 90 fish) and we reran GWAS. There was no significant change to the QTL identified in this study. Also, all the 90 fish came from hatch year, 2010. In this GWAS, we used fish from 2010 hatch year (406 fish representing 98 families) and 2012 hatch year (472 fish representing 99 families.

## Author contributions

MS, TL, and BK conceived and designed the experiments. RA-T, MS, TL, and BK performed the experiments. RA-T, AA, DL, GG, YP, BK, and MS analyzed the data. MS wrote the paper. All authors reviewed and approved the publication.

### Conflict of interest statement

The authors declare that the research was conducted in the absence of any commercial or financial relationships that could be construed as a potential conflict of interest.
